# Mitochondrial metabolism of the facultative parasite *Chilodonella uncinata* (Alveolata, Ciliophora)

**DOI:** 10.1186/s13071-023-05695-3

**Published:** 2023-03-07

**Authors:** Xia-lian Bu, Wei-shan Zhao, Wen-xiang Li, Hong Zou, Shan-gong Wu, Ming Li, Gui-tang Wang

**Affiliations:** 1 State Key Laboratory of Freshwater Ecology and Biotechnology, and Key Laboratory of Aquaculture Disease Control, Ministry of Agriculture, Institute of Hydrobiology, Chinese Academy of Sciences, Wuhan, 430072 Hubei The People’s Republic of China; 2 University of Chinese Academy of Sciences, Beijing, 100049 The People’s Republic of China; 3 Protist 10,000 Genomics Project (P10K) Consortium, Institute of Hydrobiology, Chinese Academy of Sciences, Wuhan, 430072 Hubei The People’s Republic of China

**Keywords:** Mitochondrial metabolism, Transcriptome, Adaption, Facultative parasitism

## Abstract

**Background:**

*Chilodonella uncinata* is an aerobic ciliate capable of switching between being free-living and parasitic on fish fins and gills, causing tissue damage and host mortality. It is widely used as a model organism for genetic studies, but its mitochondrial metabolism has never been studied. Therefore, we aimed to describe the morphological features and metabolic characteristics of its mitochondria.

**Methods:**

Fluorescence staining and transmission electron microscopy (TEM) were used to observe the morphology of mitochondria. Single-cell transcriptome data of *C. uncinata* were annotated by the Clusters of Orthologous Genes (COG) database. Meanwhile, the metabolic pathways were constructed based on the transcriptomes. The phylogenetic analysis was also made based on the sequenced cytochrome *c* oxidase subunit 1 (COX1) gene.

**Results:**

Mitochondria were stained red using Mito-tracker Red staining and were stained slightly blue by DAPI dye. The cristae and double membrane structures of the mitochondria were observed by TEM. Besides, many lipid droplets were evenly distributed around the macronucleus. A total of 2594 unigenes were assigned to 23 functional classifications of COG. Mitochondrial metabolic pathways were depicted. The mitochondria contained enzymes for the complete tricarboxylic acid (TCA) cycle, fatty acid metabolism, amino acid metabolism, and cytochrome-based electron transport chain (ETC), but only partial enzymes involved in the iron-sulfur clusters (ISCs).

**Conclusions:**

Our results showed that *C. uncinata* possess typical mitochondria. Stored lipid droplets inside mitochondria may be the energy storage of *C. uncinata* that helps its transmission from a free-living to a parasitic lifestyle. These findings also have improved our knowledge of the mitochondrial metabolism of *C. uncinata* and increased the volume of molecular data for future studies of this facultative parasite.

**Graphical Abstract:**

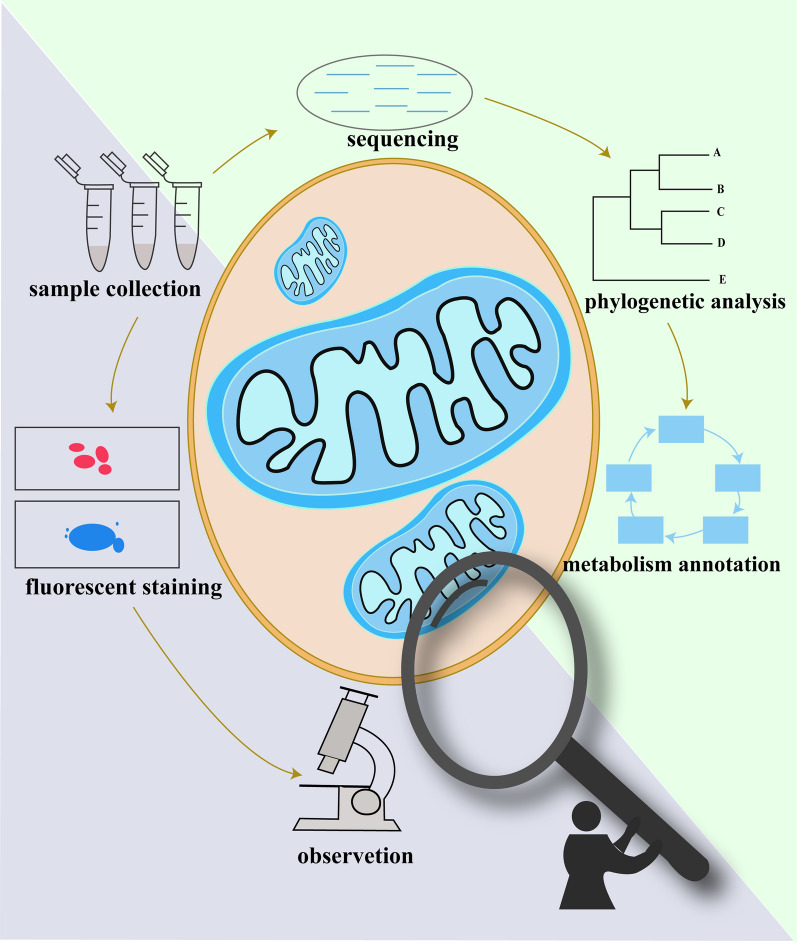

**Supplementary Information:**

The online version contains supplementary material available at 10.1186/s13071-023-05695-3.

## Background

Mitochondria are thought to have arisen as a consequence of endosymbiosis of an ancestral α-proteobacterium into an archaeon [1, 2]. Mitochondria can be categorized into five classes based on energy metabolism: aerobic mitochondria, anaerobic mitochondria, H_2_-producing mitochondria, hydrogenosomes, and mitosomes [3]. For example, anaerobic flagellates such as trichomonads have hydrogenosomes that lack an cytochrome-based electron transport chain (ETC) and can produce H_2_ [4]. The armophorean ciliate like *Nyctotherus ovalis* has a hydrogen-producing mitochondrion [5]. The amoebozoan *Entamoeba istolytica* contains mitosomes, which have lost the ability to produce ATP [6]. The free-living oligohymenophorean aerobic ciliates belonging to the genera *Tetrahymena* and *Paramecium* have more typical aerobic mitochondria [7]. These mitochondria-related organelles (MROs) have evolved several times along with the adaptation of ciliates to the anoxic and oxygen-depleted habitats [8]. The MROs of different anaerobic ciliate lineages (class Armophorea, class Plagiopylea, class Muranotrichea, etc.) have common features and possess unique characteristic as well [8–10]. Compared with these anaerobic ciliates, the understanding of mitochondrial metabolism in aerobic parasitic ciliates is still not enough.

The aerobic ciliate *Chilodonella uncinata* is considered to be a facultative parasite, which can be infective to goldfish and cause tissue damage and even host mortality [11, 12]. Other *Chilodonella* species are either free-living or obligate parasitic [13, 14]. The ability of *C. uncinata* to adapt to both free-living and parasitic environment indicates that it can use different nutrients to produce energy. Mitochondria play a central role in ATP production via oxidative phosphorylation, so it is necessary to study the mitochondria of *C. uncinata* to explore their metabolic characteristics and lifestyle adaptability. Our previous work found that there was no difference in the mitochondrial proteins between the parasitic *C. uncinata* and the free-living *C. uncinata* [15]. However, the metabolic characteristics of *C. uncinata*'s mitochondria are still unclear.

In the present study, multiple methods were combined to study the mitochondria of *C. uncinata*. First, fluorescence staining was used to observe the mitochondria in vivo. Then, the morphology of mitochondria was revealed by transmission electron microscopy (TEM). Single-cell transcriptome data of *C. uncinata* were used to identify the metabolic pathways and make the Clusters of Orthologous Groups (COG) annotation. We also sequenced the cytochrome *c* oxidase subunit 1 (COX1) gene and performed the phylogenetic analysis.

## Methods

### Specimen collection

Specimens used in this study were all collected in our laboratory since an in vitro culture and a successful infection model of *C. uncinata* had been formerly established [15]. Since the expression of mitochondrial proteins did not significantly differ between the parasitic and free-living *C. uncinata* [15], we used free-living specimens (from the culture medium) for Mito-tracker and DAPI staining, TEM, and DNA extraction for the convenience of sampling. Both free-living and parasitic *C. uncinata* were collected for the transcriptome sequencing.

### Mito-tracker and DAPI staining

Mito-tracker and DAPI stainings were carried out to locate and visualize DNA and mitochondria. First, *C. uncinata* cells were washed in sterile water, and then the Mito-tracker Red Dye was dissolved in DMSO for a stock solution. Finally, the stock solution was diluted with PBS (phosphate-buffered saline) for a working solution. *Chilodonella uncinata* specimens were subsequently stained with a few drops of the working solution for 30 min at room temperature. After the Mito-tracker Red staining, 0.02% DAPI (4′,6-diamidino-2-phenyl-indole) was added to the cell suspension for 10 min. The cells were visually inspected and photographed using Axioplan 2 imaging and Axiophot 2 (Zeiss, Oberkochen, Germany).

### Transmission electron microscopy (TEM)

For the transmission electron microscopy, *C. uncinata* cells were fixed in 2.5% glutaraldehyde. Then, the samples were postfixed in 1% (v/v) osmium tetroxide in PBS for 2 h at 4 ℃ followed by dehydration in a gradient acetone series and embedded in Araldite. Ultrathin sections were cut on a Leica Ultracut R ultramicrotome (Leica, Germany) and stained with uranyl acetate and lead citrate before being observed using a JEM-1230 Transmission Electron Microscope (JEOL, Japan).

### Transcriptome analysis and mitochondrial protein prediction

Transcriptome analysis was conducted based on our former sequencing and assembly results, with reads deposited in GenBank under the BioSample number PRJNA842862 [15]. Transdecoder v5.5.0 (http:transdecoder.github.io) was used to predict open-reading frames (ORFs). After prediction, genes were annotated using the COG database. Putative mitochondrial proteins were detected by BLASTP [16] using homologs from other ciliates and well-described mitochondrial proteomes as queries: *Homo sapiens* [17], *Saccharomyces cerevisiae* [18], and *Tetrahymena thermophila* [19]. TargetP [20] and MitoFates [21] were also used to predict the mitochondrial-targeting signals. BlastKOALA (www.kegg.jp/blastkoala/) server [22] was then used to annotate these putative mitochondrial proteins. Mitochondrial metabolic pathways were visualized with Adobe illustrator. All other visualizations were completed in R v4.1.1 [23].

### DNA extraction, amplification, and sequencing

About 500 *C. uncinata* were collected from the culture medium, suspended in lysis buffer (10 mM Tris–HCl, pH 8.0; 1 M EDTA, pH 8.0; 0.5% sodium dodecyl sulfate; 60 μg/ml proteinase K), and incubated at 55 °C for 12–20 h. DNA was extracted using the REDExtract-N-Amp Tissue PCR Kit (Sigma, St. Louis, MO, USA). The COX1 gene was amplified with two primers listed in Additional file 1: Table S1. Polymerase chain reaction (PCR) was conducted in a 20-µl reaction mixture containing 7.4 µl ddH_2_O, 10 µl 2 × PCR buffer (Mg^2+^, dNTP plus, Takara, Dalian, China), 0.6 µl of each primer, 0.4 µl recombinant Taq DNA polymerase (250 U/µl, Takara, Dalian, China), and 1 µl DNA template. PCR conditions were as follows: initial denaturation at 98 °C for 2 min, followed by 40 cycles at 98 °C for 10 s, 50 °C for 15 s, 68 °C for 1 min/kb, and a final extension at 68 °C for 10 min. PCR amplified products were sequenced on an ABI PRISM® 3730 DNA Sequencer (Applied Biosystems, USA).

### Sequence comparisons and phylogenetic analysis

BLAST [16] was used to search the homology from the NCBI database. Amino acid sequences were deduced using ExPASy translate tool (http://web.expasy.org/translate/) [24]. The secondary structure of the COX1 in *C. uncinata* was determined by PSIPRED Protein Sequence Analysis Workbench (http://bioinf.cs.ucl.ac.uk/psipred) [25] to predict coil, strand, and helix chains of the COX1 protein. The transmembrane segment prediction was conducted and visualized by the EMBOSS packages tmap and pepwheel [26]. The 3D structure of COX1 protein was predicted by Swiss-Model online server (http://swissmodel.expasy.org/interactive) [27]. Swiss-PDB viewer [28] was used to check the stereochemical quality of the structure.

Aside from the COX1 gene sequence of *C. uncinata* obtained in this study, other sequences of COX1 were retrieved from the GenBank database using PhyloSuite v1.1.15 [29]. Sequences of the class Heterotrichea were selected as outgroups. Sequence alignments, best-fit model selection, and phylogenetic tree reconstruction were conducted using plug-in programs implemented in PhyloSuite: MAFFT v7.313 [30], ModelFinder [31], MrBayes v3.1.2 [32], and IQ-TREE v1.6.8 [33]. The GTR + I + F + G4 model was selected as the best model, which was used for both maximum likelihood (ML) and Bayesian inference (BI) methods. The ML analysis was performed by the IQ-TREE v1.6.8 [33] with 1000 bootstrap replicates. BI analysis was performed using MrBayes 3.2.6 [32] with 1,000,000 generations. Trees were sampled every 1000 generations with the initial 25% of sampled data discarded as burn-in. The ML and BI trees were visualized and edited using the Interactive Tree Of Life (iTOL v4) tool [34], with annotation files generated by PhyloSuite.

## Results

### Mito-tracker and DAPI staining

**Fig. 1:**
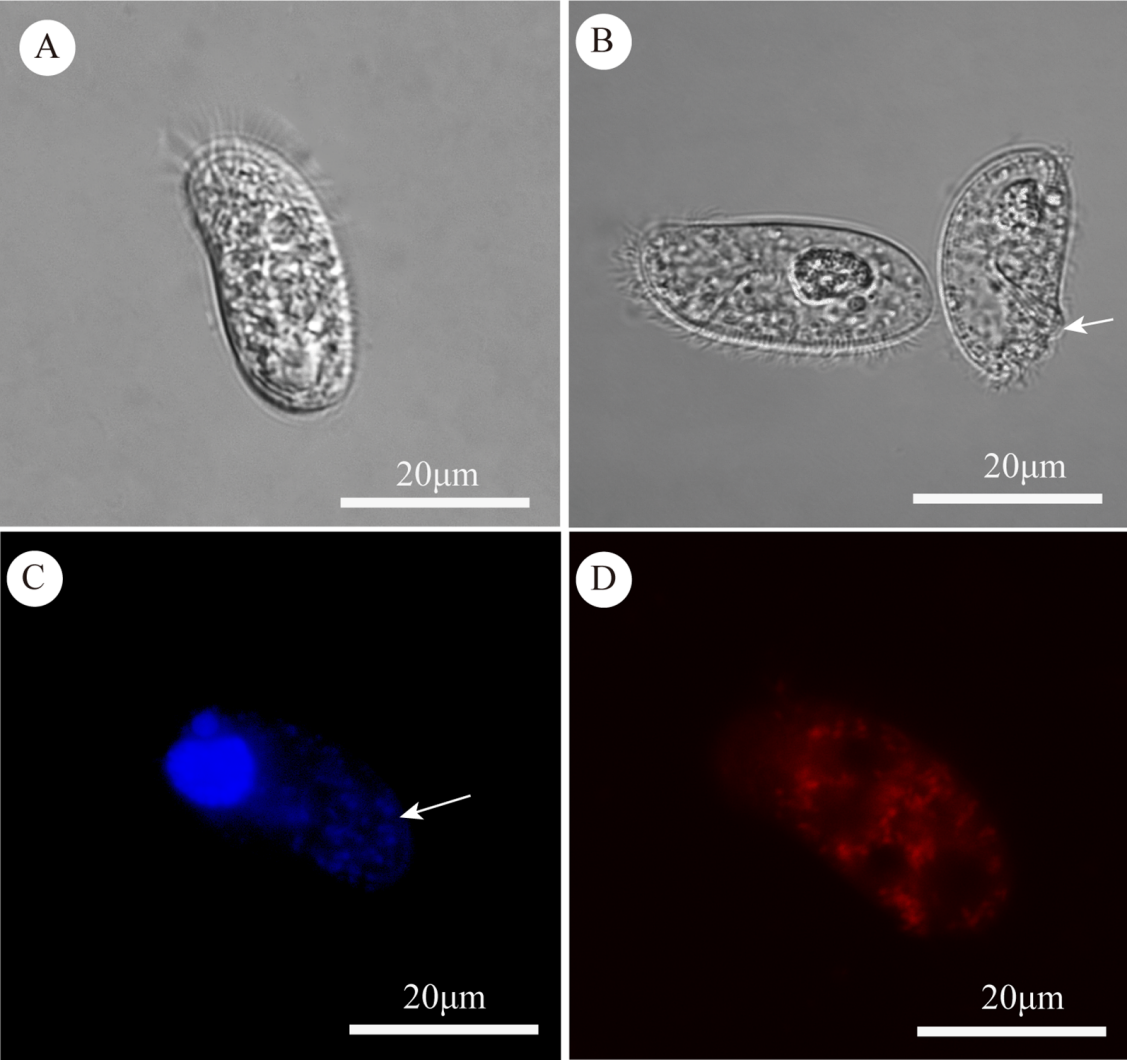
Overall morphology of *C. uncinata* and Mito-tracker Red and DAPI staining of its mitochondria. **A** Differential interference contrast (DIC) image of a living specimen from the dorsal side. **B** The macronucleus and micronucleus of a living specimen from the dorsal side (left) and cytostome (arrow) from the ventral side (right). **C** DAPI staining, showing the macronucleus and micronucleus of *C. uncinata*; arrow shows mitochondria. **D** The mitochondria of *C. uncinata* stained red with Mito-tracker Red

Different staining methods were used to observe mitochondria of *C. uncinata* in vivo. Living specimens were photographed from the dorsal side (Fig. 1A) and ventral side (Fig. 1B right) to show the gross morphology of *C. uncinata*. Cilia, cytostome, oral basket, macronucleus, and micronucleus are clearly visible. The macronucleus and micronucleus were stained blue by DAPI dye. Mitochondria were also slightly blue (Fig. 1C), which indicates that they contain traces of DNA. Mitochondria were stained red using the Mito-tracker Red staining, which revealed that they are evenly distributed within the cell (Fig. 1D).

### Transmission electron microscopy (TEM)

**Fig. 2:**
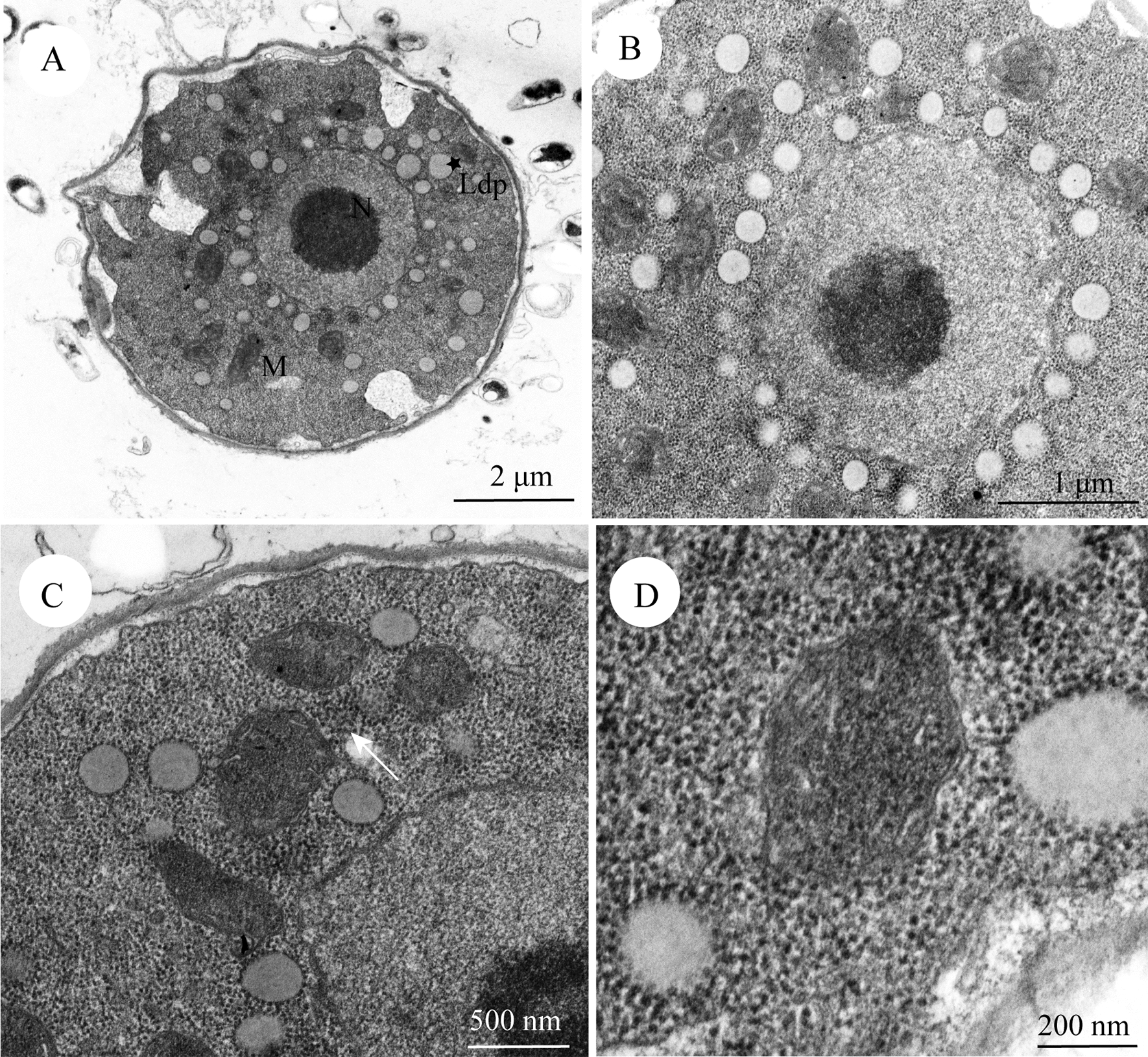
Transmission electron microscope pictures of *C. uncinata*. **A** The whole section of *C. uncinata,* where N is the nucleolus of the macronucleus, M is a mitochondrion, and the star sign denotes lipid droplets (Ldp). **B** Lipid droplets and mitochondria of various shapes are evenly distributed around the macronucleus. **C** A detail showing mitochondria and ribosomes (white arrow) around them. **D** Cristae and the double membrane of mitochondria

The macronucleus, mitochondria, and other organelles are shown in Fig. 2A. There was a circle of lipid droplets around the macronucleus, which were round in outline and had low density areas (Fig. 2B). Mitochondria were unevenly distributed among lipid droplets and their morphologies were slightly different, ranging from oval to round (Fig. 2B). Some free ribosomes were present around the mitochondria (Fig. 2C). Further magnifications allowed us to observe mitochondrial cristae and the double membrane (Fig. 2D).

### COG annotation

**Fig. 3:**
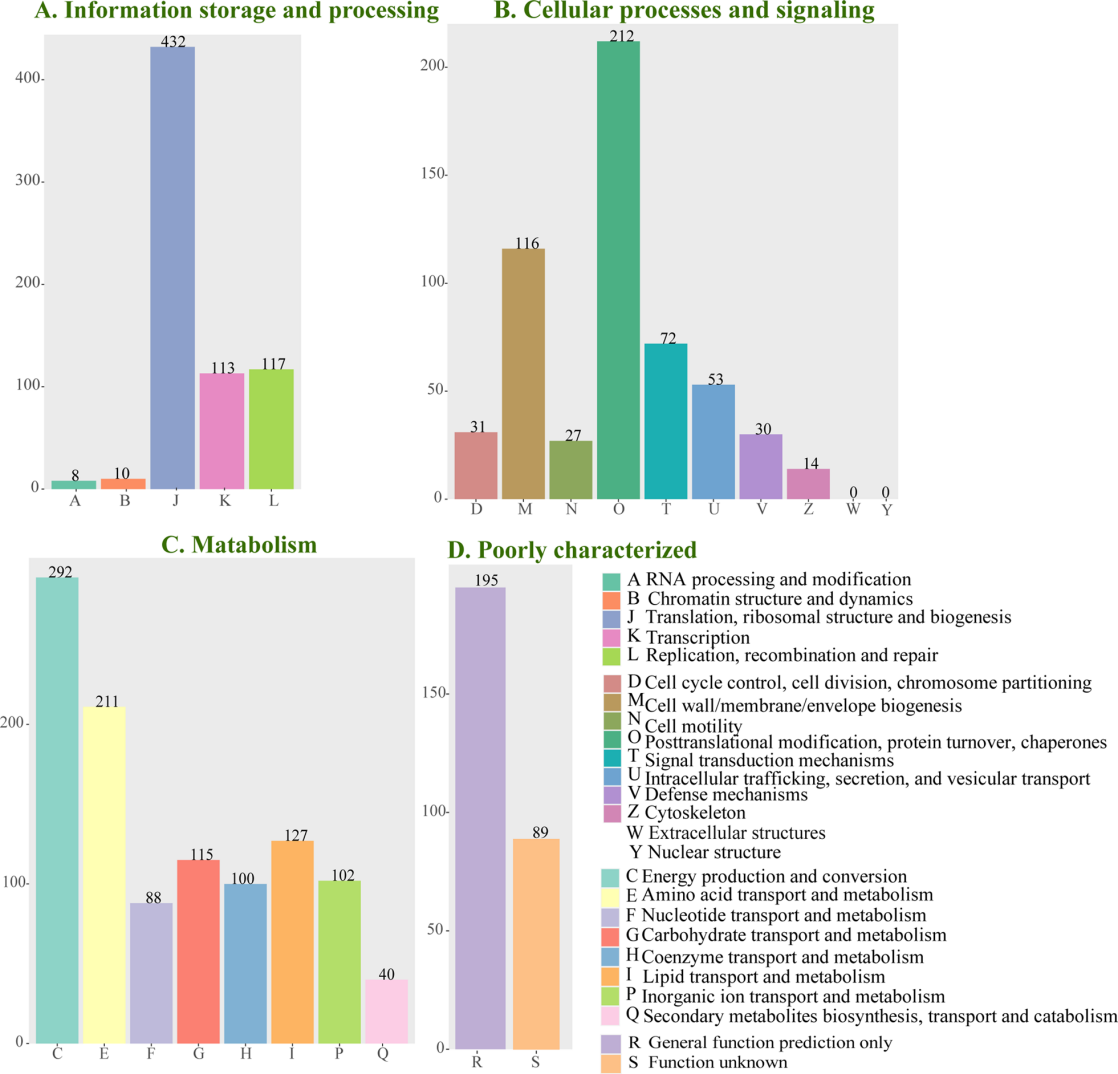
Clusters of orthologous groups (COG) annotation of unigenes. **A** Information storage and processing; **B** cellular processes and signaling; **C** metabolism; **D** poorly characterized

A total of 2594 unigenes were assigned to 23 functional classifications of COG (Fig. 3). Among them, 680 genes were assigned to the ‘information storage and processing’ category; 555 genes were assigned to the ‘cellular processes and signaling’ category; 1075 genes were assigned to the ‘metabolism’ category; 284 genes were assigned to the ‘poorly characterized’ category. Within the information storage and processing category, translation, ribosomal structure, and biogenesis were the most abundant sub-categories that accounted for 63.53% of unigenes (*n* = 432) (Fig. 3A). Within the cellular processes and signaling category, the most abundant sub-category was represented by posttranslational modification, protein turnover, and chaperones, which accounted for 38.20% of unigenes (*n* = 212). There were no assigned genes in extracellular structures and nuclear structure categories (Fig. 3B). Within the metabolism category, the top three identified functional sub-categories were energy production and conversion, amino acid transport and metabolism, and lipid transport and metabolism, which accounted for 58.60% (*n* = 630) of unigenes (Fig. 3C). As for the category named simply ‘poorly characterized,’ one of the most abundant sub-categories embraced was the general function prediction category, which accounted for 68.66% (*n* = 195) of unigenes (Fig. 3D). All these data are summarized in Additional file 1: Table S2.

**Fig. 4:**
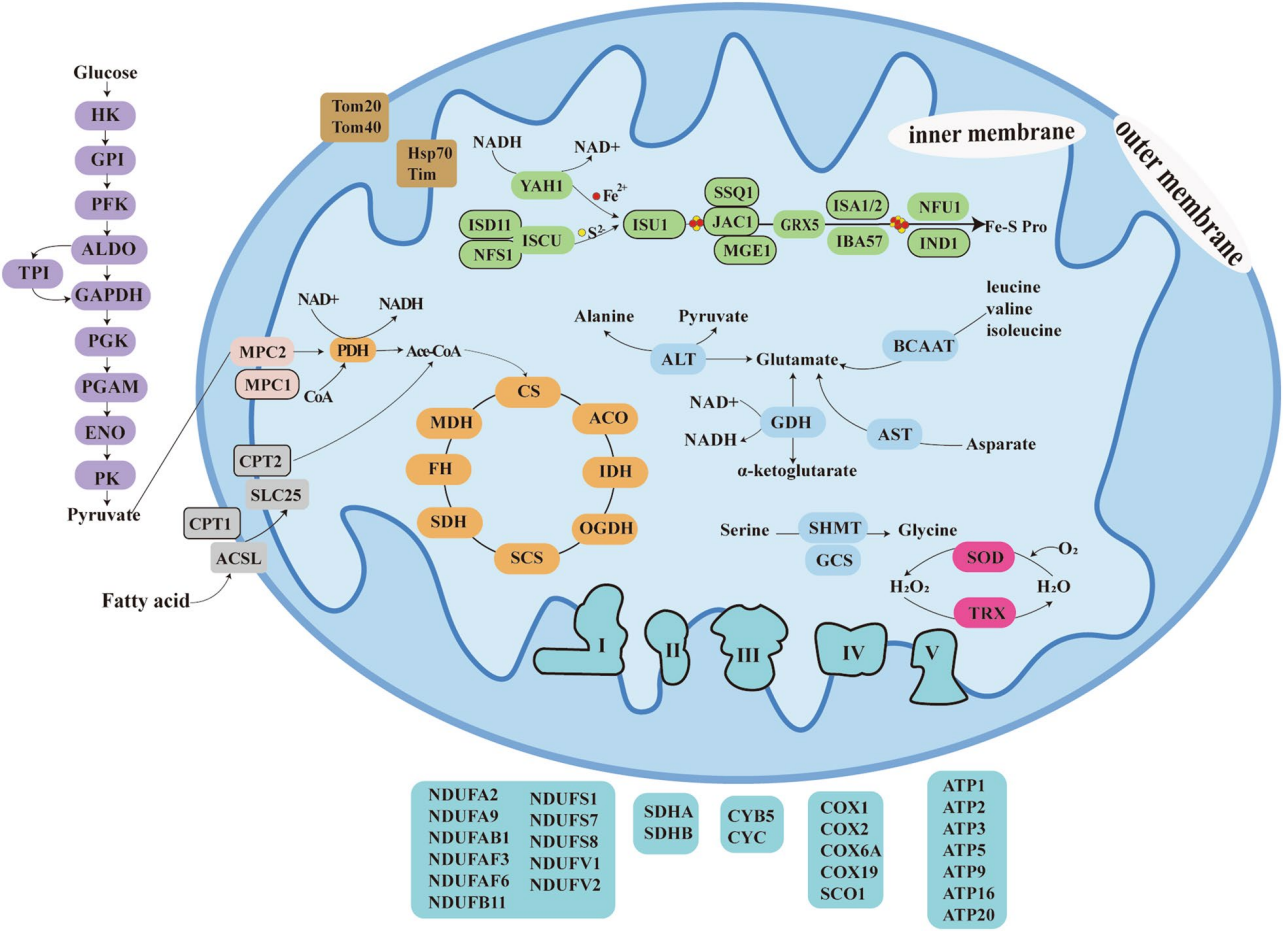
Predicted mitochondrial metabolic pathway map of *C. uncinata* based on the molecular data. Circles in different colors represent different biological functions. The subunits that were not identified are outlined by a solid line. The cycles comprise: glycolysis (purple); fatty acid metabolism (grey); mitochondrial pyruvate carrier (pink); protein import and folding (brown); iron-sulfur cluster (ISC) assembly system (green); tricarboxylic acid (TCA) cycle (yellow); amino acid metabolism (light blue); superoxide catalytic reaction (plum-red); ETC complex (dark blue). For abbreviations, see Additional file 1: Table S3

### Mitochondrial metabolic pathways

A total of 111 mitochondrial proteins were predicted based on homology to known mitochondrial proteins (Fig. 4). Among them, the number of proteins with TargetP prediction scores and Mitoprot prediction scores > 0.5 were 42 and 50, respectively. The scores represent the probability that the protein is located in mitochondria based on mitochondrial target signals (MTSs, see Additional file 1: Table S3).

Glycolysis-derived pyruvate is imported into mitochondria via the mitochondrial pyruvate carrier (MPC). Then, pyruvate is oxidized into acetyl coenzyme A (Ace-CoA) via the pyruvate dehydrogenase complex (PDH). Ace-CoA subsequently participates in the typical tricarboxylic acid (TCA) cycle. The TCA cycle includes many different enzymes, such as citrate synthase (CS), aconitate hydratase (ACO), isocitrate dehydrogenase (IDH), 2-oxoglutarate dehydrogenase (OGDH), succinyl-CoA synthetase (SCS), succinate dehydrogenase (SDH), fumarate hydratase (FH), and malate dehydrogenase (MDH). Ace-CoA can also be formed by fatty acid oxidation. First, fatty acids are activated by the acyl-CoA synthetase long-chain family (ACSL) and then transported by the solute carrier family (SLC25) and oxidized by carnitine palmitoyl transferase (CPT). Besides, glutamate is generated from different amino acids via transaminases, such as alanine aminotransferase (ALT), aspartate aminotransferase (AST), and branched-chain amino acid aminotransferase (BCAAT). Then, glutamate is transformed to α-ketoglutarate via glutamate dehydrogenase (GDH), which can join the TCA cycle when necessary.

The mitochondrial oxidative phosphorylation (OXPHOS) is completed with the help of mitochondrial ETC. A pair of electrons from NADH are donated to the co-enzyme-ubiquinone (CoQ) through the complex I. Electrons from FADH_2_ are also donated to the CoQ by the complex II. Then, these electrons are donated to cytochrome *c* by complex III, Q-cytochrome* c* oxidoreductase (also called the cytochrome reductase). Finally, cytochrome *c* transports electrons to O_2_ via the complex IV cytochrome oxidase and molecular oxygen acts as the terminal electron acceptor. In this study, 11 and 5 subunits of complex I and complex IV were identified, respectively. The subunits of complex II including the succinate dehydrogenase flavoprotein subunit (SDHA) and succinate dehydrogenase iron-sulfur subunit (SDHB) were identified. The subunits of complex III include cytochrome *b* 5 (CYB5) and cytochrome *c* (CYC). ATP1, ATP2, ATP3, ATP5, ATP9, ATP16, and ATP20 of complex V, the ATP synthase, also have been identified.

Ferredoxin (YAH1), monothiol glutaredoxin (GRX5), IBA57, and NFU1 involved in the mitochondrial iron-sulfur cluster (ISC) assembly system were also identified. The translocator of the outer membrane (TOM) and translocator of the inner membrane (TIM), which are responsible for the entry and exit of proteins, were detected as well.

### COX1 structure prediction

**Fig. 5:**
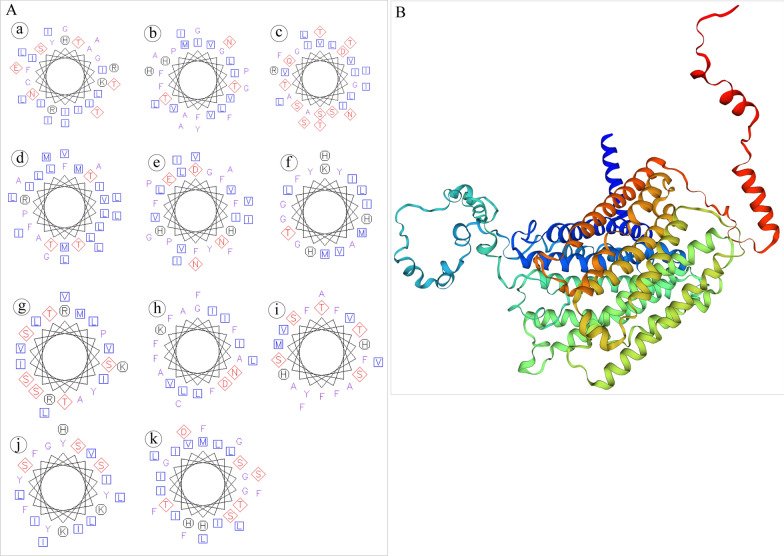
The secondary and 3D structures of COX1 of *C. uncinata* **A** There are 11 transmembrane segments in the secondary structure. Purple and blue letters represent nonpolar amino acids, while blue letters denote more hydrophobic amino acids than those in purple letters. Black letters indicate polar charged amino acids, and red letters indicate polar uncharged amino acids. **B** The 3D structure of COX1 predicted by Swiss-Model

The structural prediction of COX1 in *C. uncinata* indicated that it has 11 transmembrane segments (Fig. 5A). Among them, segments c and g have many hydrophilic polar uncharged amino acids, while the other nine segments had more nonpolar amino acids, which indicates that they are very hydrophobic. Overall, hydrophobic amino acids accounted for a large proportion of total amino acids. The predicted 3D structure of COX1 is shown in Fig. 5B. The best template identified was the COX1 of *Tetrahymena thermophila* (7w5z.7.A) with a coverage of 97% and identity of 58.82%. The Global Model Quality Estimate (GMQE) score was 0.77, and the QMEAN score was 0.68. The reliability ranges of GMQE and QMEAN are both from 0 to 1, where the higher values indicate better model quality [27].

### Phylogenetic analysis

The COX1 gene sequence obtained in this study has been deposited in the NCBI GenBank database under the accession number OP672162. The complete COX1 gene sequence comprises 1992 nucleotides and has a GC content of 24.60%. Since there is currently (September 27, 2022) no COX1 gene sequence available for the family Chilodonellidae, the most similar sequence to *C. uncinata* was the orthologue of *Tetrahymena nipissingi* under the accession number EF070295, with 52% coverage and 76.67% identity.

**Fig. 6:**
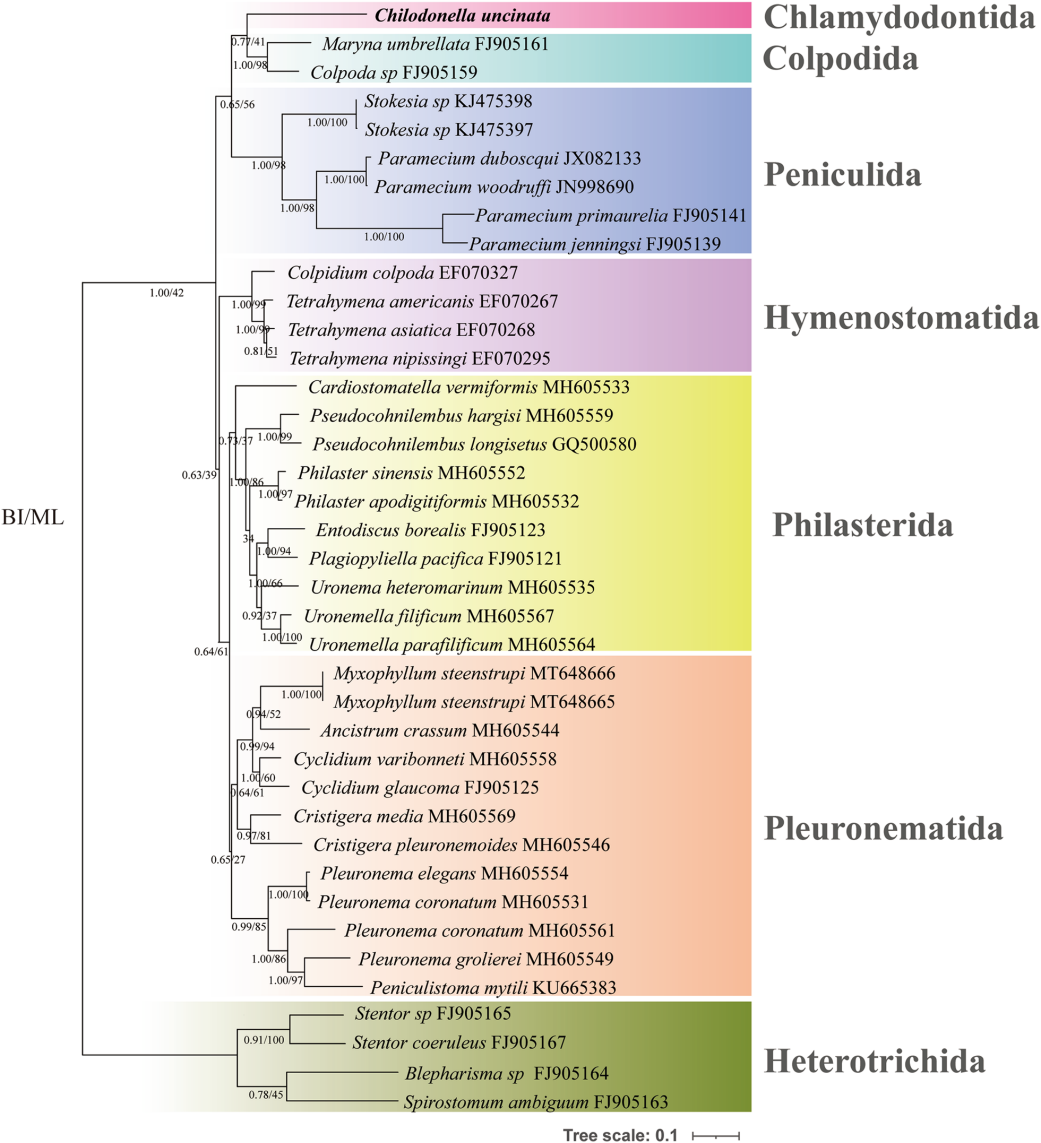
Phylogenetic trees (BI/ML) inferred from COX1 gene sequences. The newly obtained sequence is marked in bold. Numbers at nodes represent the Bayesian posterior probabilities (left) and bootstrap values (right)

The topologies generated using BI and ML algorithms were concordant, so a single consensus tree is presented herein (Fig. 6). There were no COX1 gene sequences available online of the order Chlamydodontida, so no species of this class has clustered with *C. uncinata*. It only clustered with the order Colpodida (represented by *Maryna umbrellata* and *Colpoda *sp.).

## Discussion

The mitochondria of parasitic protists evolved different shapes and sizes. The mitochondrion-related organelles (MROs) of *Blastocystis hominis* are predominantly elongate and range approximately from 1.2 to 3.0 μm in length and from 0.7 to 1.0 μm in width [35]. The hydrogenosomes of *Tritrichomonas foetus* are scattered around, and most are spherical [4]. TEM images of the marine fish parasite *Cryptocaryon irritans* showed that it had more typical mitochondria, which possess cristae structures [36]. In this study, we found that the mitochondria of *C. uncinata* are comparatively similar to those of *C. irritans* as both exhibit the cristae structures. Oxygen availability is considered the major driving force for the diversity of mitochondria [37]. Both *C. uncinata* and *C. irritans* parasitize the gills and fins of fish, where they have a sufficient supply of oxygen, while *B. hominis* and *T. foetus* parasitize the anaerobic intestinal and urogenital tracts of humans and cattle, respectively.

In this study, DAPI and Mito-tracker Red stainings were used to further confirm the existence and structure of *C. uncinata*’s mitochondria and the associated genome. The information about MROs of some common parasitic protozoa is shown in Table 1. It is worth noting that not all MROs have their own genome. Hydrogenosomes normally lack a genome, and they are entirely dependent on the nucleus for their genetic livelihood [38]. However, it has been found that the MROs of *N. ovalis* have a hydrogen-producing mitochondrial genome [5]. The flagellate *Giardia lamblia (*syn. *G. intestinalis, G. duodenalis*) living in the human small intestine has highly reduced mitochondria, called mitosomes [39]. Although we provide here the evidence of the presence of the mitochondrial genome in *C. uncinata* in this study, the complete mitochondrial genome of *C. uncinata* needs to be sequenced in the future.

**Table 1:** Mitochondria-related organelles (MROs) of parasitic protists

Parasite	Taxonomy	Host	Parasitic site	MRO	Organelle genome	References
*Ichthyophthirius multifiliis*	Alveolata, Ciliophora, Oligohymenophorea	Fish	Gills, body surface	Mitochondria	Linear	[54]
*Cryptocaryon irritans*	Alveolata, Ciliophora	Marine fish	Gills, body surface	Mitochondria	Not reported	[36]
*Chilodonella uncinata*	Alveolata, Ciliophora, Phyllopharyngea	Fish	Gills, fins	Mitochondria	Present	This study
*Nyctotherus ovalis*	Alveolata, Ciliophora, Armophorea	Cockroach	Hindgut	Hydrogen-producing mitochondria	Linear	[5]
*Trypanosoma brucei*	Discoba, Euglenozoa, Kinetoplastea	Human, cattle	Bloodstream	Mitochondria	Circular	[55]
*Trichomonas vaginalis*	Metamonada, Parabasalia, Trichomonadida	Human	Vagina	Hydrogenosome	Absent	[56]
*Leishmania*	Discoba, Euglenozoa, Kinetoplastea	Human	Macrophage	Mitochondria	Circular	[57]
*Giardia lamblia*	Excavata, Fornicata, Diplomonadida	Human	Small intestine	Mitosomes	Absent	[39]
*Cryptosporidium parvum*	Alveolata, Apicomplexa, Conoidasida	Human	Gastrointestinal	Mitosome	Absent	[58]
*Blastocystis hominis*	Stramenopiles, Bigyra	Human	Intestinal tract	Anaerobic mitochondria	Circular	[59]

With the specialization of parasite mitochondria, their metabolic pathways also changed. The mitochondrial metabolic pathways of *C. uncinata* were inferred based on single-cell RNA-seq data. The enzymes ACSL and SLC25, which are related to the fatty acid metabolism pathway, were detected on the outer and inner membrane of *C. uncinata*’s mitochondria, respectively. ACSL is crucial for fatty acid uptake and activation [40]. The member A20 of the SLC25 protein family (SLC25A20) helps with the carnitine acyl-carnitine carriage, which plays an important part in the mitochondrial β-oxidation pathway [41]. The Ace-CoA produced by β-oxidation is then added to the TCA cycle to generate energy in *C. uncinata*. Mitochondria associated with lipid droplets have increased fat oxidation, pyruvate oxidation, and electron transport [42]. In this study, many lipid droplets surrounding the mitochondria were observed by the TEM. Moreover, a large number of annotated genes were revealed to be associated with energy production and conversion. Based on the aforementioned findings, we propose that the stored lipid droplets are the main energy source for *C. uncinata* when there is no food or nutrient available in the living environment. However, different parasites store different energy substances for their use. For example, the ciliate *Balantidium ctenopharyngodoni*, inhabiting the hindgut of grass carp, stores many starch granules in the cytoplasm [43]. The intestinal trichomonad *Histomonas meleagridis* uses glycogen granules as its energy storage [44]. Various energy storage forms thus also could represent differences in the main metabolic pathways of these parasites.

In mitochondria, the oxidation of pyruvate by PDH generates Ace-CoA, which can then combine with oxaloacetate (OAA) to form citrate, the first substrate of the TCA cycle [45]. The pyruvate formed in glycolysis relies on the MPC to enter the mitochondria. The MPC is encoded by three homologous genes, MPC1, MPC2, and MPC3 in *Saccharomyces cerevisiae*, by two genes, MPC1 and MPC2, in flies, and by three genes, MPC1, MPC1-like, and MPC2, in mammals [46, 47]. In this study, only MPC1 was detected in *C. uncinata*. More validation experiments are needed in the future to prove whether MPC2 is expressed or not.

All TCA cycle-related enzymes were identified in *C. uncinata*. The TCA cycle in the mitochondrial matrix supplies NADH and FADH_2_ to the ETC, each of which donates a pair of electrons to the ETC in complexes I and II, respectively [48]. All ETC complexes are located on the inner membrane of the mitochondria [49]. In aerobic mitochondria, complexes I and II have an important role in generating ubiquinol with ubiquinone. Ubiquinone can be regenerated from ubiquinol by complex II, which is important for maintaining the activity of complex I [47]. Complex I of the free-living ciliate *Metopus contortus* appears to lack the NAD4L and NAD6 subunits [50]; complex I of *N. ovalis* and *Blastocystis* consist of 14 and 16 subunits, respectively [5]. In this study, 11 subunits of complex I are found in *C. uncinata*. SDHA and SDHB of complex II were detected as well, which is consistent with reports for *N. ovalis*, *M. contortus, and M. laminarius* [5, 9, 50]. However, only SDHA of ETC was detected in the *Plagiopyla* cf. *narasimhamurtii*, which may indicate that the ETC has lost its oxidative phosphorylation function [9]. The complex III subunits, CYB5 and CYC, were detected in *C. uncinata* in the present study, while only the Rieske protein was detected in *C. porcatum* and *M. contortus* [51]. No genes encoding complex III, IV, and V in Metopida and Clevelandellida (Armophorea) were identified, which imply that ATP synthesis via oxidative phosphorylation was lost and substituted by substrate-level phosphorylation [8].

Finally, phylogenetic analysis based on COX1 gene sequence and 3D structure prediction of its protein product confirmed the identity of this subunit. Based on these data, it can be inferred that *C. uncinata* has a relatively complete ETC system. Though ETC plays an important part in contributing to energy generation in aerobic eukaryotes, multiple studies have shown that the most highly conserved function among mitochondria and MROs is the Fe-S cluster biosynthesis [52, 53]. Fe-S clusters are essential for all organisms. The most widely distributed Fe-S cluster assembly system found in bacteria and mitochondria/MROs of eukaryotes is the ISC system [37]. In this study, the iron donor YAH1, the scaffold proteins ISCU, NFU1, and IBA57, and molecular chaperones GRX5 were detected, but the necessary catalytic cysteine desulfurase was not identified.

## Conclusions

Collectively, this is the first report of mitochondria of *C. uncinata*, studied using multiple methods. We found that *C. uncinata* has typical mitochondria with double membrane and cristae structures. Plenty of lipid droplets were found around the mitochondria, which may be the energy storage of *C. uncinata* that helps its transmission from a free-living to a parasitic lifestyle. Consistent with the morphological observations, metabolic pathways associated with fatty acid metabolism were identified based on the transcriptomic data. Except for this pathway, glycolysis, TCA cycle based on the ETC, and amino acid metabolism were also identified. The present findings identify the metabolic energy sources and cast light on the putative mechanism behind the facultative parasitism of *C. uncinata* and contribute to the prevention and treatment of chilodonelliasis in the future.

## Supplementary Information

**Additional file 1: Table S1.** Primers used in this study. **Table S2.** COG annotation results. **Table S3.** Annotation results of mitochondria metabolism.

## Data Availability

The datasets supporting the conclusions of this article are included within the article and its additional files. The COX1 nucleotide sequence generated in this study was deposited in the GenBank database under the accession number OP672162.

## Abbreviations

TEM: Transmission electron microscopy
COG: Clusters of Orthologous Genes
COX1: Cytochrome c oxidase subunit 1
TCA: Tricarboxylic acid
ETC: Electron transport chain
ISCs: Iron-sulfur clusters
ORFs: Open-reading frames
BI: Bayesian inference
ML: Maximum likelihood
